# The Impact of Racial Disparities and Social Determinants of Health on Postoperative Outcomes After Pancreaticoduodenectomy (Whipple Procedure)

**DOI:** 10.7759/cureus.91855

**Published:** 2025-09-08

**Authors:** Shivam Chandra, Vivian Liang, Anthony V Coglianese, Steven R Bonomo

**Affiliations:** 1 Surgery, A.T. Still University School of Osteopathic Medicine in Arizona (SOMA), Mesa, USA; 2 Surgery, John H. Stroger, Jr. Hospital of Cook County, Chicago, USA

**Keywords:** healthcare disparities, pancreaticoduodenectomy, postop complication, race and surgery, social determinants of health, surgical outcome, trinetx database, whipple procedure

## Abstract

Background: Pancreatectomy, particularly the Whipple procedure, is the standard surgical treatment for resectable pancreatic head tumors but carries high complication rates. Evidence suggests that race and social determinants of health (SDOH) may influence postoperative outcomes, but these disparities remain underexplored in pancreatic surgery.

Objective: This study aimed to evaluate the impact of race and SDOH on postoperative complication rates following pancreatectomy, using a large electronic health record dataset.

Methods: A retrospective cohort study was conducted using the TriNetX Research Network. Patients undergoing Whipple procedures were identified by the Current Procedural Terminology (CPT) code 48150. Postoperative complications were defined by ICD-10 codes T81 and K91. SDOH were identified using ICD-10 Z55-Z65 codes. Patients were stratified by race (White, Black, Asian, Hispanic) and SDOH status. Primary outcomes compared complication rates across racial groups. Secondary outcomes evaluated the relative risk (RR) of complications within each racial group by SDOH status, benchmarked against White patients.

Results: Among 11,546 patients, complication rates did not significantly differ between White (3649/9027, 40.4%), Black (457/1197, 38.2%), or Hispanic (349/874, 39.9%) patients, but were lower in Asian patients (157/448, 35.0%; p = 0.0234). The presence of SDOH was associated with higher complication rates across all groups. Only Black patients demonstrated a statistically significantly greater relative increase in complication risk due to SDOH compared with White patients (RR 1.52 vs. 1.51; Z = -0.19; p < 0.0001). No significant disparities related to SDOH were observed in the Hispanic or Asian groups.

Conclusions: In this multicenter analysis of pancreatectomy (Whipple procedures), complication rates were high across all racial groups, and race alone, both with and without adjustment for social determinants of health, was not a consistent predictor of outcomes. Black patients with documented SDOH had a small but statistically significant increase in complications compared to White patients with similar social risk, suggesting a modest impact that warrants further investigation. These findings highlight the importance of considering both race and SDOH when addressing disparities in surgical outcomes.

## Introduction

Pancreatic cancer (PC) is one of the 10 most commonly diagnosed cancers in the United States. In 2025, it is projected that about 67,440 new cases will be diagnosed, with an estimated 51,980 deaths expected that same year [1]. These numbers have steadily increased over the past decade, representing a 44.4% rise in incidence between 2014 and 2024 [1]. Despite progress in cancer care and treatment, pancreatic cancer remains among the most lethal malignancies [1]. Many patients still face a five-year survival rate that remains in the single digits [1,2].

When it comes to treatment, surgical resection, followed by chemotherapy and radiation, is the standard of care for those with resectable disease [3]. The most common surgical approach for tumors in the head of the pancreas is pancreaticoduodenectomy, better known as the Whipple procedure [4]. Additional indications for this procedure include chronic pancreatitis and severe pancreatic trauma [4]. Patient eligibility requires the tumor to be resectable without metastases and major vascular encasement [4,5]. While this surgery can be lifesaving, it also carries a high risk of complications. Studies have shown complication rates ranging from 30% to 60% [6], with some of the most frequent issues including postoperative bleeding, abscesses within the abdomen, pancreatic fistulas, and bile leaks [6]. 

Given how often complications occur after the Whipple procedure, it is important to recognize that surgical outcomes are influenced by more than just what happens in the operating room. Factors beyond the hospital, such as a patient’s income, education level, neighborhood, and access to healthcare, can have a significant impact on both short- and long-term recovery. A growing body of research shows that social determinants of health (SDOH) and race are closely linked to disparities in surgical outcomes and overall survival [7-9]. These structural barriers often limit access to timely diagnosis, high-quality treatment, and adequate follow-up care, which can worsen prognosis for underserved groups.

This study aims to explore how race and SDOH influence postoperative complications in patients undergoing pancreatectomy. The complications investigated in this study include ICD-10 codes T81 (complications of procedures, not elsewhere classified) and K91 (postprocedural disorders of the digestive system, not elsewhere classified). 

## Materials and methods

This retrospective cohort study was conducted using data extracted from the TriNetX Research Network [10]. The dataset included adult patients (≥18 years old) who underwent a pancreatectomy (Whipple procedure), identified using CPT code 48150, across participating healthcare organizations. Data were collected for patients who received this procedure between January 1, 2015, and December 31, 2023.

Inclusion criteria consisted of patients with a documented CPT 48150 code and available postoperative outcome data. Patients were stratified by race (White, Black, Hispanic, Asian), which was self-reported, and by the presence or absence of documented SDOH using ICD-10 Z codes Z55-Z65, which encompass factors such as educational, occupational, housing, financial, and psychosocial challenges.

Exclusion criteria included patients with missing demographic information (including race or ethnicity), incomplete outcome data, or conflicting procedural coding.

Postoperative complications were identified using ICD-10 codes T81 (complications of procedures, not elsewhere classified) and K91 (postprocedural disorders of the digestive system, not elsewhere classified). Patients were stratified by SDOH status and analyzed across racial groups.

Statistical analysis included a chi-square test to evaluate overall differences in postoperative complication rates across racial groups stratified by SDOH status. Pairwise two-proportion Z-tests were then performed to compare each group to White patients as the reference. A p-value < 0.05 was considered statistically significant. 

## Results

Table 1 shows that White patients who underwent a pancreatectomy (Whipple procedure) had a post-surgical complication rate of 40.42%, Black patients had a complication rate of 38.18% (p > 0.05), Asian patients had a rate of 35.04% (p = 0.0295), and Hispanic patients had a rate of 39.93% (p > 0.05). 

**Table 1:** Comparison of post-surgical complication rates by race after pancreaticoduodenectomy Statistical comparisons were performed using a chi-square test (χ² = 12.46, p = 0.0059) for overall differences in post-surgical complication rates across racial groups, followed by pairwise Z-tests for proportions using White as the reference group.

Race	CPT 48150	Post-surgical complications	Proportion	Pairwise p-value (vs. White)
White	9027	3649	40.42%	Reference
Black	1197	457	38.18%	0.1367
Asian	448	157	35.04%	0.0234
Hispanic	874	349	39.93%	0.7772

Table 2 shows that among patients with documented SDOH Z55-Z65, post-surgical complication rates were 59.38% for White patients, 55.36% for Black patients, 47.62% for Asian patients, and 47.76% for Hispanic patients (all p > 0.05). Table 3 shows that among patients without documented SDOH, complication rates were 39.42% for White patients, 36.41% for Black patients, 35.13% for Asian patients, and 39.28% for Hispanic patients (all p > 0.05). 

**Table 2:** Postoperative complication rates among patients with documented social determinants of health (Z55-Z65), stratified by race Statistical comparisons were performed using a chi-square test (χ² = 4.26, p = 0.235) to assess overall differences in post-surgical complication rates among racial groups. Pairwise Z-tests for proportions were subsequently conducted using White patients as the reference group. p-values for each comparison are provided in the final column. A p-value < 0.05 was considered statistically significant.

Race	CPT 48150/pancreatectomy (Whipple-type procedure)	Post-surgical complications (T81 or K91)	Proportion	Pairwise p-value (vs. White)
White	453	269	59.38%	Reference
Black	112	62	55.36%	0.4388
Asian	21	10	47.62%	0.2842
Hispanic	67	32	47.76%	0.0722

**Table 3:** Postoperative complication rates among patients without documented social determinants of health (Z55-Z65), stratified by race Statistical comparisons were performed using a chi-square test (χ² = 5.74, p = 0.1248) to evaluate overall differences in post-surgical complication rates across racial groups. Pairwise Z-tests for proportions were conducted using White patients as the reference group. p-values < 0.05 were considered statistically significant.

	CPT 48150/pancreatectomy (Whipple-type procedure)	Post-surgical complications (T81 or K91)	Proportion	Pairwise p-value (vs. White)
White	8574	3380	39.42%	Reference
Black/African American	1085	395	36.41%	0.0551
Asian	427	150	35.13%	0.0762
Hispanic/Latino	807	317	39.28%	0.9379

Table 4 compares the relative impact of SDOH on post-surgical complications following Whipple procedures across racial groups. This analysis first examines the complication risk within each racial group by comparing patients with and without SDOH and then compares these within-group differences to those observed in White patients to assess whether SDOH has a differential impact. White patients served as the reference group, with a relative risk (RR) of 1.51 for complications among those with SDOH compared to those without. Black patients had an RR of 1.52, with a ratio vs. White of 1.01 (Z = -0.19, p = 0.0001). Asian patients had an RR of 1.36 (ratio vs. White = 0.90; Z = -1.37, p > 0.05), and Hispanic/Latino patients had an RR of 1.22 (ratio vs. White = 0.81; Z = -2.11, p > 0.05). 

**Table 4:** Relative impact of social determinants of health (SDOH) on postoperative complications by race, benchmarked against White patients Statistical comparisons using a chi-square test (χ² = 16.92, p < 0.001) revealed significant differences in postoperative complication rates among patients with SDOH, stratified by race. Pairwise Z-tests, using White patients as the reference, showed significantly smaller relative increases in complications for Hispanic/Latino (p < 0.00001) and Asian patients (p = 0.035). To contextualize the findings in Table 3, we evaluated whether SDOH disproportionately impacts postoperative complication risk among racial groups undergoing Whipple procedures. This was done by first comparing complication rates within each racial group, patients with SDOH versus those without, and then benchmarking these within-group relative risks against the corresponding difference observed in White patients. The use of relative risk ratios, Z-scores, and p-values allowed for standardized cross-racial comparisons to identify where disparities are most pronounced.

Comparison	RR (with vs. without SDOH)	Ratio vs. White	Z-score	Pairwise p-value (vs. White)
White	1.51	1.00 (reference)	-	Reference
Black/African American vs. White	1.52	1.01	-0.19	0.0001
Asian vs. White	1.36	0.90	-1.37	0.24
Hispanic/Latino vs. White	1.22	0.81	-2.11	0.17

## Discussion

Racial disparities and the influence of social determinants of health on postoperative outcomes

In this study, three main findings emerged. First, overall complication rates following Whipple procedures were high across all racial groups, with only Asian patients showing a statistically significantly lower rate than White patients (p = 0.0295). Second, among patients with documented SDOH, complication rates increased across all groups, but no significant racial differences were observed. Third, when evaluating the relative impact of SDOH within each racial group, Black patients experienced a statistically significantly higher complication burden compared to White patients with similar social risk (ratio = 1.01, p = 0.0001). This suggests that SDOH may disproportionately affect surgical outcomes in Black patients, underscoring the importance of examining both race and socioeconomic context in disparity research.

High postoperative complication rates reflect surgical complexity and patient comorbidity

Table 1 displays post-surgical complication rates following Whipple procedures across racial groups. White patients experienced a complication rate of 40.42%, which was comparable to rates observed in Black (38.18%) and Hispanic (39.93%) patients; these differences were statistically insignificant (p > 0.05). In contrast, Asian patients had a significantly lower complication rate of 35.04% when compared to White patients (p = 0.0295). Overall, complication rates clustered around 40% across all groups. These findings suggest that, in this analysis, race was not a strong predictor of postoperative complications, except for Asian patients who demonstrated lower complication rates compared to White patients.

The Whipple procedure is recognized as a technically complex and demanding surgical intervention associated with high complication rates [6]. Although surgical advancements and experience at high-volume centers have reduced perioperative mortality to below 5%, complication rates remain substantial, as observed in our analysis [11-13]. This may be attributed to the procedure’s intricacy, duration, and the need for advanced perioperative and critical care support to ensure optimal outcomes [6].

Another contributing factor to the high complication burden is the underlying health status of patients undergoing this procedure. More than half of Whipple candidates have am Age-Adjusted Charlson Comorbidity Index (ACCI) score of 3 or higher, indicating a moderate-to-severe burden of chronic illnesses and age-related risk factors [14]. An ACCI score ≥ 3 reflects the presence of multiple comorbidities and/or advanced age, which elevates the preoperative risk for postoperative complications and adversely impacts both short- and long-term outcomes [15].

Postoperative outcomes stratified by social determinants of health and race

Table 2 examines post-surgical complication rates among patients with documented SDOH undergoing Whipple procedures. White patients had a complication rate of 59.38%, compared to 55.36% in Black patients, 47.62% in Asian patients, and 47.76% in Hispanic patients. All comparisons yielded p-values > 0.05, indicating no statistically significant differences. These findings suggest that, among patients with adverse SDOH, captured using ICD-10 Z codes Z55-Z65, which include factors such as education, employment, housing instability, financial hardship, social environment, and psychosocial stressors, minority groups did not experience worse surgical outcomes compared to White patients.

Table 3 assesses complication rates among patients without documented SDOH and similarly found no statistically significant differences across racial groups. Complication rates were 39.42% in White patients, 36.41% in Black patients, 35.13% in Asian patients, and 39.28% in Hispanic patients (all p > 0.05).

These results differ from prior studies, including the work by Alwatari et al., who reported that race and ethnicity were independently associated with adverse postoperative outcomes [16]. In contrast, our findings suggest that race alone was not an independent determinant of postoperative complications. One potential explanation for this discrepancy is the difference in study design: whereas prior research focused primarily on 30-day complication rates, our analysis encompassed complications occurring at any point postoperatively, allowing for a broader and more longitudinal assessment of outcomes.

Importantly, our study accounted for SDOH using structured ICD-10 Z codes, enabling more precise adjustment for socioeconomic and psychosocial risk factors. Compared to earlier studies such as those by Alwatari et al. and Tavakkoli et al., our methodology allowed for a more nuanced understanding of the role of race in surgical outcomes [16,17]. Once SDOH were accounted for, race was not consistently associated with increased complication risk following pancreatectomy. Furthermore, across both SDOH-affected and non-SDOH groups, minority patients did not experience significantly worse outcomes compared to White patients.

Impact of SDOH on postoperative outcomes 

Table 4 evaluates the relative impact of SDOH on postoperative complications following Whipple procedures across racial groups. The analysis first assessed complication risk within each racial group by comparing patients with and without documented SDOH and then benchmarked these within-group relative risks against White patients to assess for differential impact. Notably, this was the only analysis in which a minority group, specifically Black patients, demonstrated significantly worse outcomes compared to White patients. The RR of complications among Black patients with SDOH versus those without was 1.52, yielding a cross-racial ratio of 1.01 when compared to White patients (RR = 1.51), with a statistically significant difference (p < 0.0001). In contrast, the corresponding ratios for Hispanic and Asian patients were lower and statistically insignificant (p > 0.05).

Although unadjusted complication rates among SDOH-affected patients appeared similar across racial groups (Table 2), the within-group comparison in Table 3 indicates that SDOH may confer a disproportionately greater risk of complications for Black patients. Black patients with documented SDOH experienced a greater relative increase in postoperative complications compared to Black patients without SDOH, and this increase was more pronounced than the corresponding difference observed among White patients.

Although this finding reached statistical significance, the magnitude of the difference was minimal. The primary conclusion of this study is that, when evaluating racial disparities in Whipple procedures through multiple analyses using the TriNetX database, no clinically meaningful differences were identified, indicating comparable outcomes across racial groups.

Strengths and limitations

This study leverages a large, real-world, multicenter dataset from the TriNetX Research Network, providing a broad and demographically diverse sample of patients undergoing Whipple procedures. TriNetX allows researchers to access aggregated, de-identified electronic health record data across multiple healthcare organizations, enabling a comprehensive understanding of patient demographics, diagnoses, and outcomes in routine clinical practice. By incorporating ICD-10 Z codes to capture SDOH, the analysis offers a structured and quantifiable approach to evaluating socioeconomic and psychosocial risk factors. Unlike prior studies focused on 30-day outcomes, this study examines postoperative complications over a longer horizon, offering a more comprehensive assessment of long-term surgical outcomes [6]. Additionally, the stratified analyses by race and SDOH provide a nuanced view of how social context intersects with clinical outcomes.

The retrospective design of the study limits the ability to infer causality between race, SDOH, and surgical outcomes. The use of administrative codes, particularly ICD-10 Z55-Z65 for SDOH, may lead to underreporting or misclassification due to inconsistent documentation practices. Complication data were analyzed cumulatively without differentiation by severity or timing, which may obscure specific postoperative risk periods. Furthermore, key confounding variables such as hospital volume, surgeon experience, and perioperative care protocols were not available in the dataset. Lastly, findings may not be fully generalizable beyond the institutions included in the TriNetX network, given potential regional variations in care delivery and documentation. Additionally, cancer staging, a critical prognostic factor influencing postoperative outcomes, was not available in the TriNetX database and could not be adjusted for in this analysis. The inability to control for stage-specific disease severity may confound the interpretation of racial and SDOH-related differences in complication rates. 

## Conclusions

This study underscores the complex relationship between race, SDOH, and postoperative outcomes following Whipple procedures. While aggregate complication rates were similar across most racial groups, Black patients experienced a significantly greater relative increase in complications when SDOH were present, compared to their White counterparts. These findings suggest that the intersection of race and social risk factors contributes to disproportionate surgical morbidity, particularly in socioeconomically disadvantaged Black patients. By leveraging structured ICD-10 Z-code data within a large national cohort, this study offers one of the first cross-racial analyses of SDOH-related surgical disparities in pancreatic surgery. These results emphasize the urgent need for improved perioperative risk stratification, targeted support interventions, and equity-focused care models that account for non-clinical barriers to recovery.

## Appendices

Supplemental material

**Table 5:** CPT and ICD-10 codes for pancreatectomy and associated postoperative complications and social determinants of health

Codes	Description
CPT 48150	Pancreatectomy (Whipple procedure)
ICD-10 T81	Complications of procedures, not elsewhere classified
ICD-10 K91	Postprocedural disorders of the digestive system, not elsewhere classified
ICD-10 Z55-Z65	Social determinants of health (education, occupation, housing, economic, and psychosocial)
